# High-Performance On-Chip Racetrack Resonator Based on GSST-Slot for In-Memory Computing

**DOI:** 10.3390/nano13050837

**Published:** 2023-02-23

**Authors:** Honghui Zhu, Yegang Lu, Linying Cai

**Affiliations:** Key Laboratory of Photoelectric Materials and Devices of Zhejiang Province, Faculty of Electrical Engineering and Computer Science, Ningbo University, Ningbo 315211, China

**Keywords:** von Neumann architecture, computational efficiency, power consumption, photonic in-memory computing, phase change material

## Abstract

The data shuttling between computing and memory dominates the power consumption and time delay in electronic computing systems due to the bottleneck of the von Neumann architecture. To increase computational efficiency and reduce power consumption, photonic in-memory computing architecture based on phase change material (PCM) is attracting increasing attention. However, the extinction ratio and insertion loss of the PCM-based photonic computing unit are imperative to be improved before its application in a large-scale optical computing network. Here, we propose a 1 × 2 racetrack resonator based on Ge_2_Sb_2_Se_4_Te_1_ (GSST)-slot for in-memory computing. It demonstrates high extinction ratios of 30.22 dB and 29.64 dB at the through port and drop port, respectively. The insertion loss is as low as around 0.16 dB at the drop port in the amorphous state and about 0.93 dB at the through port in the crystalline state. A high extinction ratio means a wider range of transmittance variation, resulting in more multilevel levels. During the transition between crystalline and amorphous states, the tuning range of the resonant wavelength is as high as 7.13 nm, which plays an important role in the realization of reconfigurable photonic integrated circuits. The proposed phase-change cell demonstrates scalar multiplication operations with high accuracy and energy efficiency due to a higher extinction ratio and lower insertion loss compared with other traditional optical computing devices. The recognition accuracy on the MNIST dataset is as high as 94.6% in the photonic neuromorphic network. The computational energy efficiency can reach 28 TOPS/W, and the computational density of 600 TOPS/mm^2^. The superior performance is ascribed to the enhanced interaction between light and matter by filling the slot with GSST. Such a device enables an effective approach to power-efficient in-memory computing.

## 1. Introduction

With the high requirement in terms of efficient energy and high computing force in big data applications, current electronic computing systems are approaching their limit in computing performance due to a von Neumann bottleneck (that is, the blockage of information flow caused by the separation of memory and computing units) which affects the processing speed of the computer and increases additional losses [1]. Therefore, it is imperative to explore novel calculation methods. Photonic in-memory computing technology which removes the distance between the computing and memory units has received great attention owing to its high speed and high bandwidth. However, traditional photonic computing devices [2,3,4] are volatile so the continuously consuming-energy supply is required to maintain the current state. To decrease power consumption, novel functional materials are employed to design photonic devices with the advantages of non-volatility and tunability. Particularly, chalcogenide phase change materials (PCM) have been widely used in integrated photonics due to their unique characteristics such as non-volatile polymorphisms and high optical contrast among them [5,6,7,8].

Though great effort has been made to develop photonic phase-change computing devices, there are challenges in terms of extinction ratio (ER) and insertion loss (IL) [1,9,10,11,12]. Most studies are based on the device structures which cover the chalcogenide PCM directly on the top of the waveguide or microring resonators. The change in optical transmission of the device can be modulated by controlling the polymorphism of the PCM. Such structure suffers from the weak interaction between light and PCM, and consequently, the modulation efficiency is relatively low, increasing power consumption. Zhang et al. [13] proposed a wavelength-selective 2 × 2 optical switch based on PCM-assisted microring resonators. The ERs at the through and drop ports are ~20 dB, and the additional losses at the through and drop ports are about 0.9 dB and 2 dB, respectively. To enhance ER, a 1 × 2 tunable switch based on microring resonators was proposed by using a Ge_2_Sb_2_Te_5_ (GST)-embedded Si waveguide [14]. The ERs at the through port and drop port achieved 25.57 dB and 18.75 dB, respectively. However, the large absorption coefficient of crystalline GST leads to a high IL of the switch. The ILs at the through port and the drop port are about 1.95 dB and 5.04 dB, respectively. To decrease the IL, a 1 × 1 optical switch was proposed by using a GSST-assisted silicon racetrack resonator [15]. The IL of the amorphous state is 0.19 dB, and the ER at the resonance wavelength is ~18 dB. A relatively low IL and high ER can be both obtained by using the slot waveguide filled with PCM due to the enhancement of the light-matter interaction [16,17].

As the most widely used PCM, GST has a small bandgap and produces inter-band absorption in the telecommunication frequency band, resulting in a high loss in both states [18]. A low-loss PCM GSST is obtained by replacing a part of Te with Se in the atomic structure of GST. The amorphous and crystalline refractive indexes (n_a-GSST_ = 3.4 + 0.00018i and n_c-GSST_ = 5.1+ 0.42i [19]) are lower for GSST in comparison with GST (n_a-GST_ = 3.94 + 0.045i and n_c-GST_ = 6.11 + 0.83i [20]). It leads to a lower IL for GSST-based photonic devices. The increased bandgap of the GSST is favorable for improvement in ER; therefore, GSST is employed in this study.

Compared with bare waveguide, slot waveguide facilitates the interaction between light and matter, and thus improves the modulation efficiency. In comparison with a microring resonator, a racetrack resonator has the advantages of a large coupling length and high coupling efficiency [21]. Combining the slot waveguide and racetrack resonator can capitalize on their respective advantages to improve ER and IL. However, few studies have been devoted to investigating such a desirable structure. In this paper, a 1 × 2 racetrack resonator based on GSST-slot is proposed for photonic in-memory computing. By filling the slot with GSST, the interaction between PCM and light is increased, and the modulation efficiency is improved. The IL is as low as around 0.16 dB at the drop port in the amorphous state, and about 0.93 dB at the through port in the crystalline state. The ERs at the through port and drop port achieve about 30.22 dB and 29.64 dB, respectively. Such high ER provides substantial room for transmittance variation, supporting more multilevel levels. During the amorphous-to-crystalline transition, the tuning range of the resonant wavelength (RW) is as high as 7.13 nm, which plays an important role in the realization of reconfigurable photonic integrated circuits. The proposed device demonstrates scalar multiplication, which is crucial for photonic computing cells to realize complex computing tasks. The scalar multiplication of a single phase-change unit can be extended to the matrix-vector operation by using multiple phase-change units. It also demonstrates neuromorphic computing with an accuracy of 94.6% on the recognition MNIST dataset, which is 1.6% lower than that of devices with ideal characteristics. High ER provides a wider range of weights and more multi-level weights, which are crucial for efficient neuromorphic in-memory computing. The computational energy efficiency of the device can reach 28 TOPS/W, which is higher than that of previously demonstrated devices, and the computational density of a single computational unit can reach 600 TOPS/mm^2^. The superior performance of the device is approaching more efficient in-memory computing.

## 2. Device Principle and Parameter Optimization

### 2.1. Device Design and Scalar Multiplication Concepts

**Figure 1:**
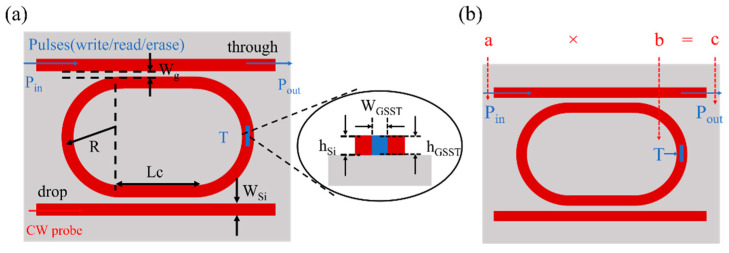
(**a**) Device structure of a racetrack resonator based on GSST-slot. A bidirectional pump-probe scheme can be employed for write/erase and readout operations. Inset is a cross-sectional view along the GSST-Si slot waveguide. (**b**) Scalar multiplication of two numbers a × b = c, mapping numbers a and b to the read pulse P_in_ and transmittance T of the device, respectively.

Figure 1a shows the 1 × 2 GSST-slot racetrack resonator structure on the silicon-on-insulator (SOI) platform, which consists of two strip waveguides, a racetrack resonator, and GSST filled in the slot. When the GSST is in the crystalline state, the coupling between the input waveguide and the resonator does not meet the phase-matching condition due to the high absorption of the GSST, and the light is directly output from the through port. When the GSST is in the amorphous state, the phase-matching condition is satisfied, and the light is coupled into the resonator at the resonant wavelength. By controlling the crystallization degree of the GSST, multi-level tuning of the device transmission can be achieved. A bidirectional pump–probe scheme was employed for GSST amorphization by write pulse (P_write_) and the crystallization by erase pulse (P_erase_). The state of the GSST is read out by the optical transmission through a read pulse (P_in_). To avoid the influence of P_in_ on the state of the GSST, it is necessary to satisfy E_Pin_ < E_0_ < E_Pwrite_, where E_Pwrite_ and E_Pin_ are the respective energies of the write pulse (P_write_) and the read pulse (P_in_). E_0_ is the threshold energy for the crystalline GSST to begin amorphization.

Due to the existence of many intermediate states in the phase transition process of GSST, corresponding to different refractive indices, the light will be attenuated to different degrees when passing through the waveguide, resulting in distinguishable optical transmission signals, which can encode information [10]. Figure 1b shows a schematic diagram of the scalar multiplication of a × b = c, where the numbers a, b, and c are mapped to the input read pulse (P_in_), the device transmittance T, and the output pulse (P_out_), respectively. Here, T—which can be modulated by the write pulse (P_write_)—correlates to the coupling between the electromagnetic mode propagating in the waveguide and the GSST in the slot [22]. Varying the size of the read pulse (P_in_) can change the output pulse (P_out_) at the through port to realize the scalar multiplication operation.

### 2.2. Parameter Optimization

**Figure 2:**
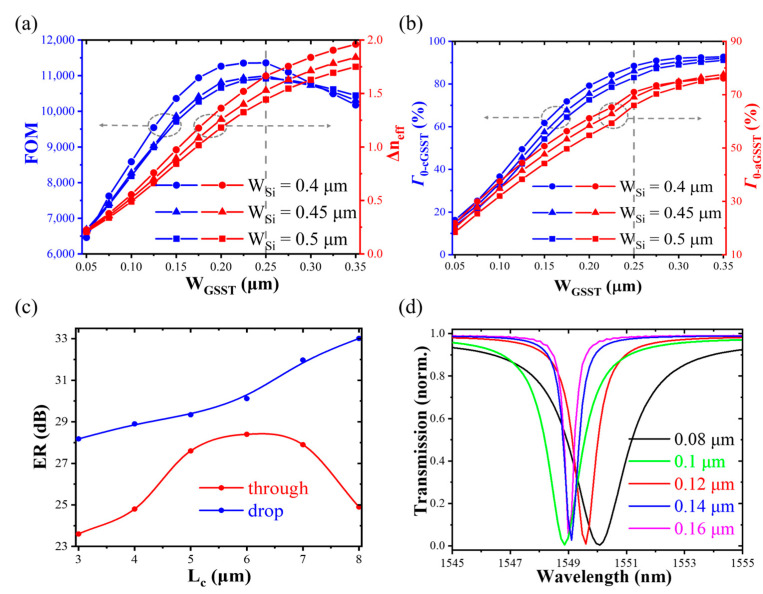
(**a**) Dependence of FOM and Δ*n*_eff_ on GSST-slot width. (**b**) *Γ*_0_ versus GSST-slot width in the crystalline and amorphous states. (**c**) ER versus the coupling length. The relevant parameters of the device are set as *W_g_* = 0.15 μm, *W_Si_* = 0.45 μm, *L_GSST_* = 1 μm, and *W_GSST_* = 0.25 μm. (**d**) The coupling gap versus the transmittance of the device in the amorphous state.

The relevant parameters of the device selected are as follows: a resonator radius of *R* = 5 μm, GSST height of *h_GSST_* = 0.22 μm, and silicon waveguide height of *h_Si_* = 0.22 μm. The optical quality factor FOM = $\Delta n_{\mathrm{eff}}/k_{a-n\mathrm{eff}}$ is introduced to determine the width of the slot [16], where ∆*n*_eff_ represents the difference in the real part of the effective refractive index of the hybrid GSST-Si slot waveguide between the crystalline (cGSST) and amorphous (aGSST) state, and *k*_a-neff_ is the imaginary part of the effective refractive index in the amorphous state. To support the fundamental mode propagation the conventional waveguide widths of 0.4 μm, 0.45 μm, and 0.5 μm are selected. The GSST width *W_GSST_* varies from 0.05 μm to 0.35 μm. As shown in Figure 2a, ∆*n*_eff_ increases with the GSST-slot width. This is because the *n*_eff_ of cGSST greatly varies, while the *n*_eff_ of aGSST has a small change due to the relatively small difference in refractive index between aGSST and silicon. Therefore, when GSST is in an amorphous state, the optical mode of the hybrid waveguide is very similar to that of the silicon waveguide. In contrast, there is a big difference in the crystalline state [16]. FOM reaches the maximum when *W_GSST_* = 0.25 μm due to the strong interaction between PCM and light.

The power confinement factor, which represents the ratio of the optical field power in the slot region to the power in the entire waveguide region, is an important parameter for the confinement of the optical field by the slot waveguide. Here, the optimal slot width can be further determined by obtaining the power confinement factor *Γ*_0_ in the slot region [23]:

(1) $$\Gamma 0=\frac{\iint_{\Omega GSST}{\left|E(x,y)\right|}^{2}dxdy}{\iint_{\Omega Si}{\left|E(x,y)\right|}^{2}dxdy}$$

where *E(x, y)* is the electric field vector, *Ω_GSST_* is the slot region, and *Ω_Si_* is the entire waveguide region. As shown in Figure 2b, regardless of GSST states, *Γ*_0_ increases with the GSST width. The distribution of optical modes in the slot area increases with the increase of the slot area, thus improving the optical field power in the slot area. After *W_GSST_* exceeds 0.25 μm, the optical field power in the slot region is gradually close to that in the hybrid waveguide, making the *Γ*_0-aGSST_ and *Γ*_0-cGSST_ (GSST in amorphous and crystalline states) slowly change. By comprehensively considering FOM and *Γ*_0_, the slot width *W_GSST_* is selected to be 0.25 μm. When *W_Si_* = 0.45 μm, *Γ*_0-aGSST_ and *Γ*_0-cGSST_ achieve as high as 69.7% and 86.2%, respectively, which are much larger than the previously reported *Γ*_0_ for directly covering the PCM on the surface of the silicon waveguide (3.9% and 6% in the amorphous and crystalline states, respectively [24]), indicating that the slot region has a strong optical field confinement effect.

To determine the coupling length (*L_c_*), the ER (ER = $10\log(T_{\max}/T_{\min})$, where *T*_max_ and *T*_min_ represent the maximum and minimum transmittance of the same port in two states, respectively, of the device is used to optimize it. As shown in Figure 2c, as *L_c_* increases, the ER of the through port first increases and then decreases. It reaches a maximum value in the case of *L_c_* = 6 μm due to occurance of the critical coupling in the amorphous state. When GSST is in a crystalline state, the input waveguide and resonator do not meet phase-matching. As *L_c_* increases, the coupling effect gradually degrades. Thus, *L_c_* is designed to be 6 μm.

The coupling gap (*W_g_*) of the device is determined according to the through port transmittance of the device at the resonant wavelength in the amorphous state. As shown in Figure 2d, the device is overcoupled when *W_g_* = 0.08 μm and 0.1 μm. When *W_g_* = 0.12 μm, the resonance peak of the device deeply drops, close to the critical coupling state. Therefore, the coupling gap of *W_g_* = 0.12 μm is designed.

**Figure 3:**
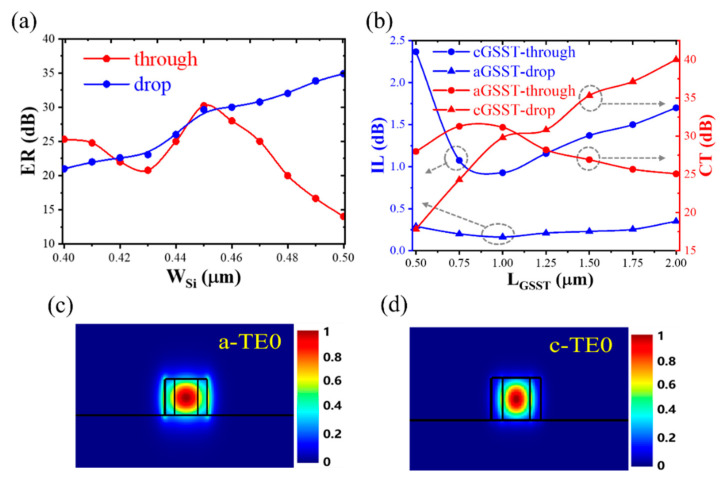
(**a**) ER versus the waveguide width. (**b**) IL and CT versus GSST length. TE0 mode distributions in the (**c**) amorphous and (**d**) crystalline states.

To obtain a high ER, the waveguide width (*W_Si_*) is optimized. As shown in Figure 3a, with the increase of the *W_Si_*, the ER of the through port first increases and then decreases, and the ER of the drop port consistently increases. When the GSST is in the amorphous state, the input waveguide and resonator meet the phase-matching at *W_Si_* = 0.45 μm. The ER of the through port reaches the maximum value of ~30.22 dB, and the drop port also has a larger ER of ~29.64 dB, which is higher than the devices based on PCM-on-top racetrack resonator previously reported [15]. Therefore, *W_Si_* is selected to be 0.45 μm in this study.

The GSST length (*L_GSST_*) was further optimized by monitoring the IL and crosstalk (CT). IL means how much light is transmitted through the corresponding active port and CT represents the partial light through the inactive port. When GSST is in the amorphous state, light is mainly output from the drop port. As *L_GSST_* increases (see Figure 3b), the IL of the drop port first decreases and then increases.In the case of *L_GSST_* = 1μm, the input waveguide and the resonator meet the phase-matching. The light at the drop port reaches a maximum, indicating the minimum of the IL and the maximum of the CT. The minimum IL of the drop port is about 0.16 dB, and the CT of the through port achieves 31.15 dB. When GSST is in the crystalline state, insufficient *L_GSST_* leads to partial light leaking to the drop port, and excessive *L_GSST_* will increase the absorption of the material (high absorption in the crystalline state). In the case of *L_GSST_* = 1 μm, the IL of the through port is at a minimum value of 0.93 dB, and the CT of the drop port is larger (~29.8 dB). The ILs of both crystalline and amorphous states are smaller than the devices based on the PCM-on-top microring resonator previously reported [13]. Therefore, *L_GSST_* is optimized to be 1μm. The IL of the through port is larger than that of the drop port. This is because the coupling length is long enough to enable more optical signals to be coupled into the racetrack resonator. In addition, the slot structure increases light absorption due to the enhanced light–matter interaction. Figure 3c,d show the TE0 mode distributions of GSST in the amorphous and crystalline states, respectively, where *W_Si_* = 0.45 μm, *W_GSST_* = 0.25 μm, and *h_GSST_* = 0.22 μm. When GSST is crystalline, the optical field is mainly confined in the slot due to the high refractive index of GSST.

## 3. Device Simulation

### 3.1. Switching Simulation

**Figure 4:**
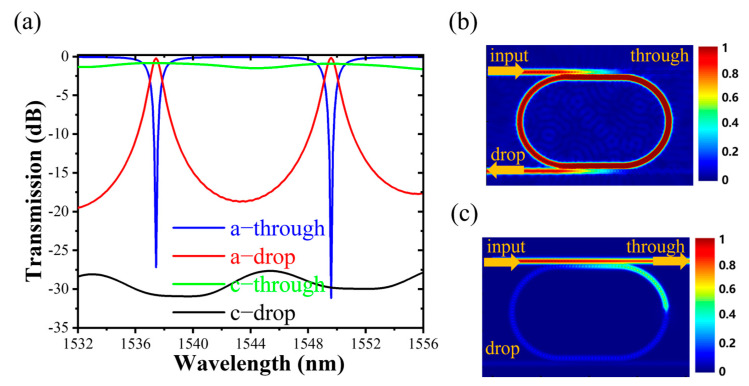
(**a**) Spectral responses at the through and drop ports of the device. The normalized electric field distributions of GSST in (**b**) amorphous and (**c**) crystalline states (*λ_res_* = 1549.6 nm).

Based on the optimized geometry (*W_g_* = 0.12 μm, *L_c_* = 6 μm, *W_Si_* = 0.45 μm, *W_GSST_* = 0.25 μm, *L_GSST_* = 1 μm) of a 1 × 2 GSST-slot racetrack resonator photonic device, the spectral response is simulated by Lumerical Solution’s 3D-FDTD [25], as shown in Figure 4a. Compared with the microring resonator with the same radius, the racetrack resonator photonic device has a smaller free spectral range (FSR) due to the increase in the circumference of the racetrack resonator. The FSR of the device is around 12.17 nm, about 4.37 nm smaller than that of a microring resonator with the same radius (*R* = 5 μm). There is a modal mismatch between the straight waveguide part and the ring part of the racetrack resonator, and the ring radius is relatively small, resulting in a bit increase in resonator loss [26]. Therefore, the resonant peak is not as sharp as the microring resonator, and its quality factor (Q) is smaller than that of the microring resonator. It is noted that GSST has a tunable range of device RW up to ~7.13 nm during the switching between crystalline and amorphous states, providing a choice for realizing reconfigurable photonic integrated circuits [14]. Figure 4b,c are the normalized electric field distributions of the device in amorphous and crystalline states at *λ_res_* = 1549.6 nm, respectively. In the crystalline state, the phase-matching conditions are not satisfied. The high absorption due to the high refractive index of GSST blocks the transmission of optical signals in the resonator.

### 3.2. Thermal Simulation

To study the energy consumption for the write/erase operation, a three-dimensional finite element model was established by COMSOL to simulate the temperature change of GSST in the slot. The solid heat transfer and electromagnetic wave modules are used in the model. The material parameters set for the simulation are listed in Table 1. The phase transition of GSST is a thermodynamic transformation process. The amorphization process is to directly heat the GSST temperature by an optical write pulse to above the melting point temperature (T_m_ = 819 K), and then rapidly quench to obtain a disordered amorphous state. The crystallization process requires heating the GSST temperature above the glass transition temperature (T_g_ = 413 K) but below the melting point temperature, and then annealing to form an ordered structure.

**Figure 5:**
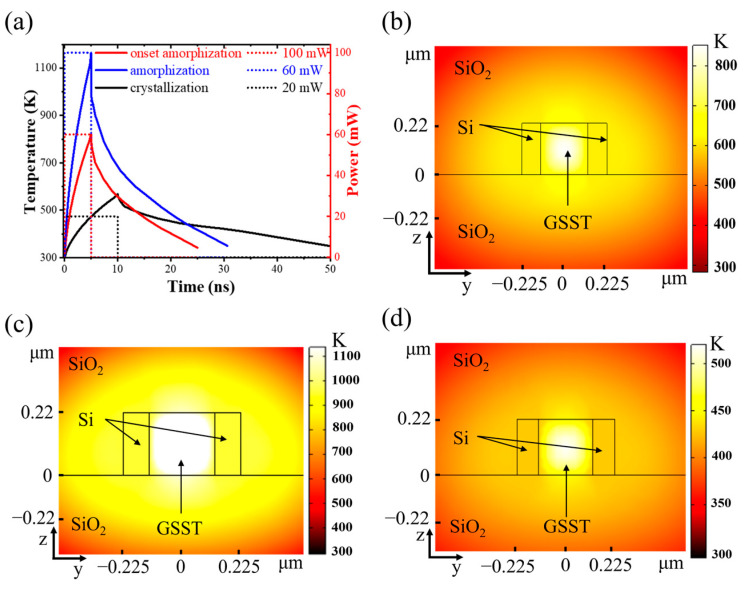
(**a**) Temperature response of GSST and input optical pulse; 2D cross-sectional temperature distribution at the slot for GSST (**b**) onset amorphization, (**c**) complete amorphization, and (**d**) crystallization.

Figure 5a shows the temperature response under a write optical pulse of 60 mW–5 ns. It makes the temperature of GSST reach its melting point (around 819 K), at which GSST onset amorphization. The two-dimensional (2D) cross-sectional temperature distribution (see Figure 5b) confirms the central area at the slot being heated above its melting point. The corresponding energy of 300 pJ is assumed to be the threshold energy (E_0_). The GSST can be completely amorphized by a write optical pulse with an amplitude of 100 mW and a width of 5 ns (see Figure 5a). As shown in Figure 5c, the whole area in the slot is heated above its melting point, suggesting that the full amorphization can be completed after quenching to room temperature. To complete the full crystallization, the erase optical pulse is required to heat GSST over the crystallization temperature (about 413 K) and below the melting point. An optical pulse with an amplitude of 20 mW and a width of 10 ns can meet this requirement. As shown in Figure 5d, the whole GSST area exhibits a temperature distribution between 413 K and 819 K, indicating that full crystallization can be done.

Table 2 compares some performance parameters of this study with previously reported phase-change devices based on microring resonators. The proposed device in this study demonstrates the larger ER and smaller IL at the drop port. It also has improved performance in terms of device size and write/erase pulse energy.

## 4. Scalar Multiplication

To describe the influence of the proportion of crystalline volume on the refractive index during the phase transition process of GSST, a crystallization level parameter F is introduced. The effective dielectric constant *ε_eff_ (λ)* of such GSST films can be estimated using the effective dielectric theory [30]. There are several popular effective-medium expressions, including the Lorentz–Lorenz, Bruggeman, and Maxwell–Garnett approximations [31]. Bruggeman and Maxwell Garnett expressions are deduced from an aggregate model and a coated-sphere configuration, respectively. Considering the characteristics of the collection of point-like polarizable atoms, it is approximate to calculate the *ε_eff_ (λ)* of PCMs through the Lorentz–Lorenz effective-medium expression [32]:

(2) $$\frac{\varepsilon_{\mathrm{eff}}-1}{\varepsilon_{\mathrm{eff}}+2}=F\times \frac{\varepsilon_{c-\mathrm{GSST}}\;-1}{\varepsilon_{c-\mathrm{GSST}}\;+2}+(1-F)\times \frac{\varepsilon_{a-\mathrm{GSST}}\;-1}{\varepsilon_{a-\mathrm{GSST}}\;+2}$$

where *ε_c-GSST_* and *ε_a-GSST_* represent the dielectric coefficients of GSST in crystalline and amorphous states, respectively. The relationship between the dielectric coefficient and the refractive index is $\;\varepsilon \left(\lambda \right)={\left(n+k\cdot i\right)}^{2}$ Through the F, the transmittance T of the device can be obtained. Then, the scalar multiplication can be carried out by inputting the low-energy read pulse (P_in_) and monitoring the change of the output pulse (P_out_) at the through port.

**Figure 6:**
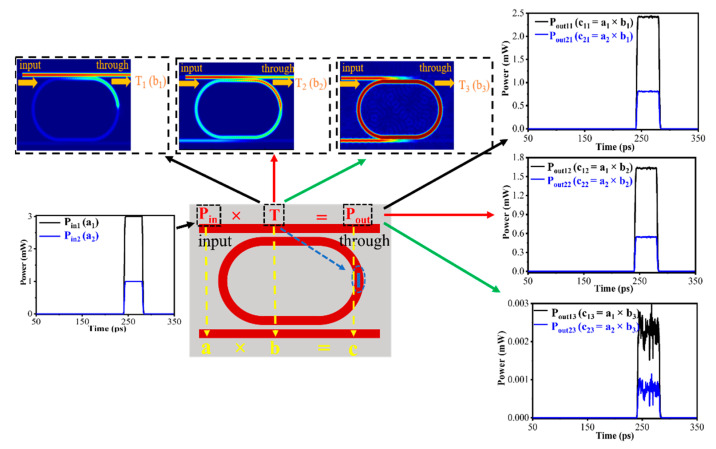
Schematic diagram of the scalar multiplication P_in_ × T = P_out._ Two read pulses P_in1_ (black curve) and P_in2_ (blue curve) with different amplitudes are input into the devices with different GSST states representing different T. The corresponding outputs (P_out_) can be obtained by measuring the device transmission.

An erase pulse of 20 mW–10 ns makes GSST fully crystallize, and the transmittance at the through port is T_1_ = 0.81. The optical pulses with different power, e.g., P_in1_ (black curve) and P_in2_ (blue curve) with amplitudes of 3 mW and 1 mW, respectively, are coupled into the straight waveguide in sequence. The corresponding output pulses P_out11_ and P_out21_ at the through port can be obtained, as shown in Figure 6. That is to say, the scalar multiplication of the P_in_ × T = P_out_ is carried out. By employing a write pulse of 60 mW–5 ns, the device is driven to the intermediate state and the transmittance of the through port is T_2_ = 0.54. The output pulse also satisfies scalar multiplication (the output curve is P_out12_ and P_out22_). To fully amorphized GSST, a write pulse of 100 mW–5 ns is applied to the device. The corresponding transmittance T_3_ at the through port is approximately 0. The output pulse curves are P_out13_ and P_out23_, which also satisfy the scalar multiplication relationship a × b = c (where a represents the input pulse P_in_, b represents the transmittance T, and c represents the output pulse P_out_). Here, a, b, and c can be normalized to [0, 1] for encoding. A single phase change unit can be extended to multiple phase change units, resulting in matrix-vector operations, which are the foundation of most modern computing fields, such as artificial intelligence.

## 5. Recognition Test

In addition to scalar multiplication calculations, our devices can be used in photonic neural networks. In photonic neural networks, the higher the extinction ratio of the device, the wider the range of weights obtained, resulting in more multi-level weights and lower dynamic power consumption to achieve precise weights [33].

**Figure 7:**
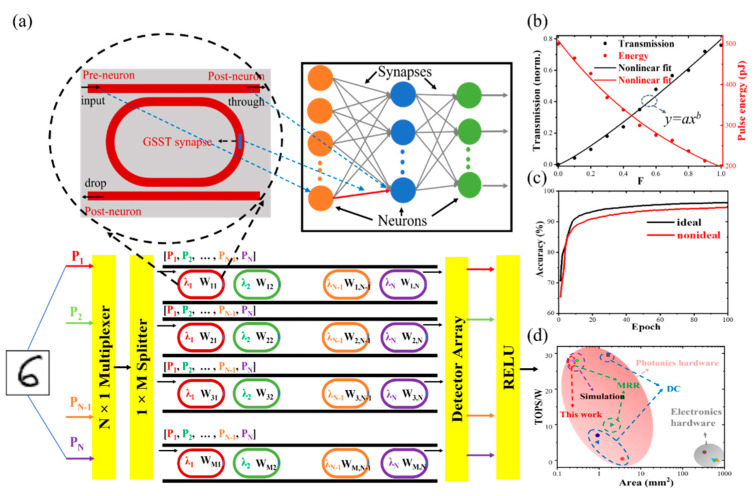
(**a**) Schematic diagram of the device for neural networks. (**b**) Through-port transmittance and energy as a function of crystallization levels F (where a = 0.8, b = 1.18). (**c**) Accuracy on MNIST dataset using ideal characteristics and nonideal characteristics of the GSST-slot racetrack resonators. (**d**) Comparison of footprint and computing energy efficiency between this work and previously reported devices including microring resonator (MRR) and directional coupler (DC).

As shown in Figure 7a, the device input and output waveguides act as pre-neurons and post-neurons, and the PCMs act as synapses for modulating signals. The MNIST dataset containing tens of thousands of images was employed for training and testing [34]. The size of the image is fixed to 28 × 28 pixels. A simple error backpropagation (BP) neural network was built for the training task, where the numbers of neurons in the input layer, hidden layer, and output layer are 784, 200, and 10, respectively. Gradient descent is used to update the weights and biases so that the loss function is continuously reduced, thereby improving the accuracy. The RELU function is chosen as the neuron activation function, which maps the neuron’s input from 0 to 1. The number of training epochs and learning rate for the dataset are 100 and 0.1, respectively. The performance of the device is tested by recognizing the handwritten dataset. To input 784 pixels of the dataset into the M × N synaptic array, the pixels are input into the first layer of neurons in the form of pulses, and the corresponding weights are used to do dot-product operations. The additions are then collected, and then are transmitted to the neurons of the next layer through the activation function, storing the weights of the next layer. After multiple passes, digital recognition is achieved. Since the FSR of the device is about 12 nm, there can be eight non-interfering resonant wavelengths in the wavelength range of 1500 nm to 1600 nm, setting N to 8. Figure 7b shows the transmittance T at the through port under different crystallinity (F). The fitted curve has good linearity in comparison with previously reported work on slot-ridge waveguides with PCMs [33]. Note that the transmittance change curve approaching linear can improve the recognition accuracy [35]. The T corresponds to the weight in the network structure, which is mapped to the photonic synapse to perform the corresponding matrix multiplication operation. Figure 7c shows the accuracy of the dataset based on GSST-slot racetrack resonators. After training 100 times, our device with nonideal characteristics has an accuracy rate of up to 94.6%, which is 1.6% lower than that of devices with ideal properties. The accuracy of our device is higher than previously reported [33,36]. The high accuracy and low loss resulting from the large weight range of the device provide a new solution for efficient neuromorphic computing.

We evaluated the computing energy efficiency of the device. When the crystallization level (F) changes from 0.3 to 1, the change in its transmittance tends to be linear (see Figure 7b). The transmittance of this part (0.2 to 0.78) is selected and mapped to the weights of the synaptic array. The corresponding maximum IL and minimum IL are ~7 dB and ~1.1 dB, respectively. Assuming a single-wavelength channel input power of 10 mW, the average power consumption of a single device is about 5.15 mW. When the array size is 128 × 8, the power consumption of the array is 5.27 W. Assuming that the data sampling rate is 18 Gbit/sec [37] and 8-channel multiplexing is used, the calculated energy efficiency is 18 × 10^9^ × 128 × 8 × 8 × 1/5.27 × 10^−12^ = 28 TOPS/W. The area of a single device is 240 μm^2^, and the calculated density is about 600 TOPS/mm^2^. As shown in Figure 7d, the footprint and computing energy density of this work are in comparison with previously reported work [8,37,38,39,40]. It can be seen that this work has the potential to demonstrate greater computing energy efficiency in a smaller area.

## 6. Fabrication Method

**Figure 8:**
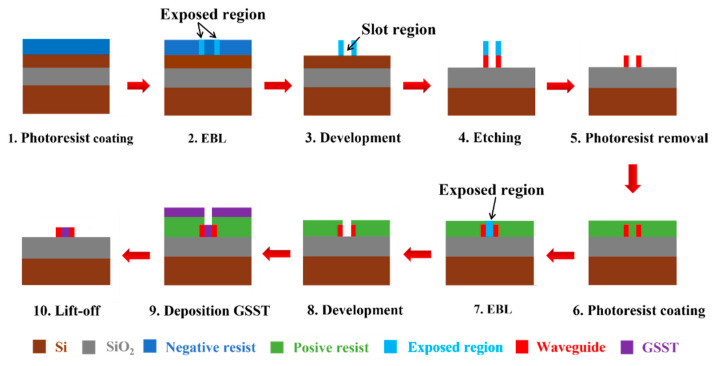
The suggested fabrication process.

Figure 8 shows the suggested fabrication process, which can be described as follows. First, a negative photoresist is spin-coated on the surface of a clean SOI wafer and then lithography is performed by an electron beam lithography (EBL). After development, the waveguide is etched by inductively coupled plasma reactive ion etching (ICP-RIE) using C_4_F_8_ and SF_6_ mixed gas. Because the height of the slot is the same as the height of the waveguide, only one etching is required to prepare a slot waveguide. After removing the photoresist on the surface of the SOI wafer, the positive photoresist is spin-coated. EBL is used to expose the slot region, and after development, GSST in the slot region is deposited by using a magnetron sputter. After the photoresist is removed, the desired structure can be obtained.

## 7. Conclusions

In conclusion, we propose a racetrack resonator based on GSST-slot for in-memory computing. The modulation efficiency is improved by embedding the GSST into the waveguide. Simulation results show that the device has a high extinction ratio of 30.22 dB at the through port. The drop port has a low insertion loss of 0.16 dB in the amorphous state. The phase transition process of GSST at the slot is thermally simulated, and the input read pulse energy E_Pin_ is less than 300 pJ. The low IL and high ER enable scalar multiplication with a wider computational range. It can also be extended to matrix-vector operations for a photonic neuromorphic network. In the photonic neuromorphic network, the recognition accuracy on the MNIST dataset is 94.6%, which is 1.6% lower than that of devices with ideal characteristics. The computational energy efficiency reaches 28 TOPS/W. The computational density of a single computational unit is up to 600 TOPS/mm^2^. The high performance exhibited by the proposed device provides a new approach to more efficient photonic in-memory computing.

## Figures and Tables

**Table 1:** Relevant material parameters used for simulation.

Material	*C_p_* (J/kgK)	*K* (W/mK)	*ρ* (kg/m^3^)
aGSST [27]	213	0.19	5870
cGSST [27]	199	0.57	6270
Si [20]	720	149	2330
SiO_2_ [20]	740	1.38	2200

**Table 2:** Performance comparison of the phase-change devices based on microring resonators.

Device Type	PCM Size (μm^3^)	Resonator Circumference (μm)	ER-Through/ ER-Drop (dB)	IL-Through/ IL-Drop (dB)	RW Shift (nm)	E_write_/ E_erase_ (pJ)
GST disk/ Si_3_N_4_-microing [28]	0.25 × 0.785 × 0.01	-	13.64/ 21.25	0.46/ 0.75	~1	200/-
GST pad/ Si-racetrack [29]	3 × 1.5 × 0.02	37.4	12.36/-	2.5/-	0.2	3600/ 900
GST embed/ Si-microing [14]	1 × 0.48 × 0.02	31.4	25.57/ 18.75	1.95/ 5.04	4.63	1063/ 181
GSST pad/ Si-racetrack [15]	15 × 0.3 × 0.045	92.8	18/-	0.19/-	~0	-
GSST slot/ Si-racetrack (this study)	1 × 0.25 × 0.22	43.4	30.22/ 29.64	0.93/ 0.16	7.13	500/ 200

## Data Availability

Data underlying the results presented in this paper are not publicly available at this time but may be obtained from the authors upon reasonable request.
